# Longevity of the Sexes

**Published:** 1892-07

**Authors:** 


### LONGEVITY OF THE SEXES.

We extract the following sensible remarks from a lengthy article by Gertrude Hammond Harper, M. D., of Spring Valley, N. Y., a near neighbor to one of the editorial staff of this journal, although as we are informed, personally unknown to each other:

Regarding longevity of women as compared with men, my experience teaches that the comparison not only is favorable, but that women come out ahead of the men. Averaging the families I know, I have met with more old women than old men, and whence comes the old adage: "If a widower wishes a second wife let him hold out his hand and he will find a widow clinging to each finger." Surely a woman has to lose her husband to become a widow. I know of many women who have been ailing and complaining during the last twenty-five or thirty years, but who yet hold out bravely. Why? Because (all the fashionable abuses notwithstanding) woman's tenacity of life is greater than man's. Woman may be compared with the willows that bend during the storms, but after the blasts are over they again stand erect; while the men may be likened to the oaks that snap or break.

Behold women who have reared eight or ten or more children, who all day long attend to the wants of the family and nurse a babe at night, perchance not once in the year having an undisturbed night's rest, yet she drags on and on, having a smile for her husband whenever he expects one. Again there are many women who are sickly and ailing from following the foolish fashions that frivolous men and women create, among which tight lacing and the weight suspended below the hips are the most fruitful sources. If male evil-doers were sentenced to wear for one month the clothing of fashionable women and be compelled to keep on their feet all day, they would with one accord pronounce themselves more severely punished than by imprisonment, and they would speedily become ill. I have seen this tried and know whereof I speak. But this only proves woman's superior powers of endurance. If women in general can once be made to see their folly, and if men on the other hand be made to understand that it is in their own interest to desist from admiring wasp-like waists, driving women to tight lacing to please the men; if the men did not in their sickly sentimentality worship lily-white faces and flabby soft white hands, young women would not be so much afraid to let the sun shine on their faces and apply their hands to domestic duties that would impart tone to the muscles. On the whole men are most to blame for women's infirmities, and if the above follies were corrected women would soon outstrip men in physical endurance *on the long run.*

I consider it a part of the mission of women physicians to labor indefatigably to remedy these gross evils; they should point out at every opportunity the importance of giving each internal organ sufficient room to perform its function; pointing out where each organ is situated, the size of it and its function. In very few years the result would be so apparent and gratifying as to bring the insurance risks rather in favor of woman instead of against her, as is the case at present.

